# Parameter inference for stochastic reaction models of ion channel gating from whole-cell voltage-clamp data

**DOI:** 10.1098/rsta.2024.0224

**Published:** 2025-03-13

**Authors:** Luca Del Core, Gary R. Mirams

**Affiliations:** ^1^ Centre for Mathematical Medicine & Biology, School of Mathematical Sciences, University of Nottingham, Nottingham NG7 2RD, UK

**Keywords:** parameter inference, uncertainty quantification, state-space models, stochastic reaction networks, differential moment equations, ion channel gating

## Abstract

Mathematical models of ion channel gating describe the changes in ion channel configurations due to the electrical activity of the cell membrane. Experimental findings suggest that ion channels behave randomly, and therefore stochastic models of ion channel gating should be more realistic than deterministic counterparts. Whole-cell voltage-clamp data allow us to calibrate the parameters of ion channel models. However, standard methods for deterministic models do not distinguish between stochastic channel gating and measurement error noise, resulting in biased estimates, whereas conventional approaches for stochastic models are computationally demanding. We propose a state-space model of ion channel gating based on stochastic reaction networks, and a maximum likelihood inference procedure to estimate the unknown parameters. Simulation studies show that: (i) our proposed method infers the unknown parameters with low uncertainty and outperforms standard approaches whilst being computationally efficient, and (ii) considering stochastic mechanisms of flickering between conducting and non-conducting open states improves the estimates in the total number of ion channels. Finally, the application of our method to experimental data correctly distinguished the 50-Hz measurement error from noise due to stochastic gating. This method improves data-driven models of ion channel dynamics, by accounting for stochastic gating and measurement errors during inference.

This article is part of the theme issue ‘Uncertainty quantification for healthcare and biological systems (Part 1)’.

## 1. Introduction

**Figure 1.**
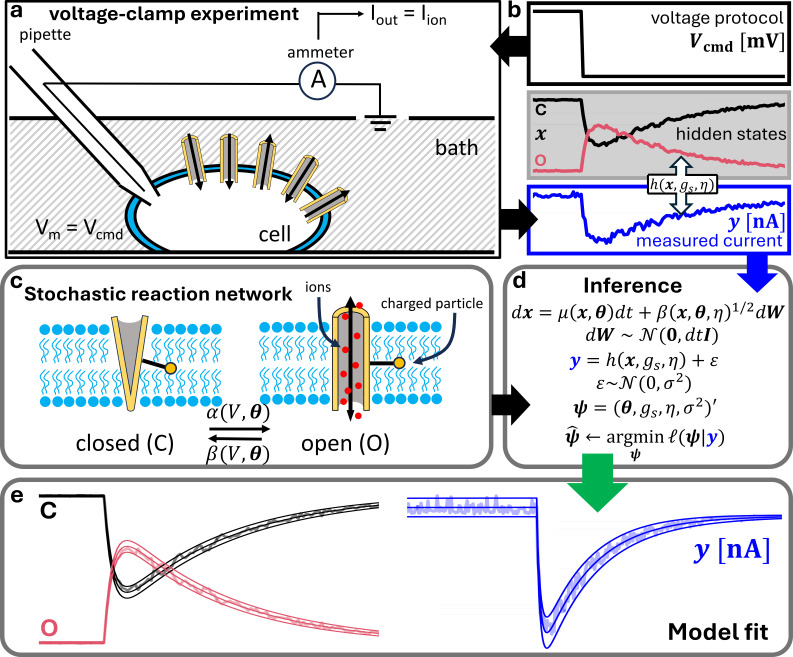
(a): a cell in solution with its membrane voltage $V_{\text{m}}$ clamped to a voltage protocol $V_{\text{cmd}}$ that may change over time (b, top). As $V_{\text{m}}$ varies, the channels change their conformation xx, e.g. Closed (C) – Open (O) (b, middle), thus increasing or decreasing the flow of ions through them. The ionic current $y\;y=I_{ion}$ (b, bottom) is measured with a voltage-clamp amplifier acting as a membrane current ammeter. The stochastic model of ion channel gating is written in a state-space formulation (d), where the dynamics are described by stochastic reaction networks, as shown in plot panel (c) for the simplistic case of a C–O model. The unknown parameters are estimated by optimizing the log-likelihood (d), and the resulting first two moments of xx and yy are returned, as displayed in plot panel (e) for a C–O model. The second moment of xx and yy is shown as 95% confidence interval (upper and lower lines) around the mean (middle line).

The electrical activity of cells is regulated by proteins residing in their membrane, such as ion channels, exchangers and pumps [1]. Small disparities between the net charge of solutions either side of the membrane result in a potential difference, called the transmembrane or membrane potential [2]. The membrane potential changes as different ionic currents flow into and out of the cell. Many of these currents are carried by a specialized family of proteins called voltage-gated ion channels that form a pore in the membrane, allowing the passage of certain types of ions. *Voltage-gated* means that changes in membrane potential cause conformational changes of these ion channel proteins, which allow ions to cross the membrane if they are in an open conformation, while preventing flow when they are closed [1]. This mechanism is graphically represented in figure 1c for the case where an ion channel has only two possible configurations, open (O) or closed (C). Single-channel experiments based on patch-clamp techniques showed that independent trial replicates of steps to the same voltages produced different random patterns of ion channel gating [3]. This stochasticity results in fluctuations of individual ionic conductances [4] and can have important effects on the electrical dynamics of the whole cell [5,6]. For example, in neuronal cells, the stochastic behaviour of ion channel gating can affect some electrical properties of the cell, such as the firing threshold [7] and spike timings [8]. Furthermore, in cardiac myocytes simulations of random channel gating induced variability in the duration of consecutive APs [9,10], termed beat-to-beat variability.

These findings inspired the development of stochastic models to describe the mechanisms underlying ion channel gating [11]. Despite their differences, each formulation describes the transitions of channels between a finite set of possible configurations (states), that happen at rates which depend on the membrane potential [12]. The probability that an ion channel is in a particular state at a certain time $t_{k}$ is assumed to depend only on the previous state at time $t_{k-1}$. Thus, for a collection of channels of size η, the dynamics of transition between their configurations can be described by a discrete-state continuous-time Markov chain [13]. This memoryless process has a probability distribution P that follows a partial differential equation (PDE) called the chemical master equation (CME), which is the standard formalism to describe stochastic reaction networks [14]. In general, the CME has no explicit solution, but different realizations of the stochastic process can be simulated using the Gillespie algorithm [14]. However, this algorithm is computationally expensive because at each iteration one has to wait for the time when the next reaction is triggered, and thus many modifications exist, such as the tau-leaping algorithm [15].

These computational limitations have led to the increasing popularity of using stochastic differential equations (SDEs) to approximate the distribution of state occupancies for the true discrete-state continuous-time Markov chain describing ion channel gating. For example, a first attempt was made by Fox & Lu [16], who extended the deterministic Hodgkin–Huxley model by incorporating a noise term into the equations describing stochastic gating. Although this approach computationally outperforms the traditional Markov-chain formulation in several model settings [17], it has been shown that the solutions of this type of SDE deviates from the true discrete-state Markov chain model [17–19], and therefore several modifications have been proposed to overcome this limitation. For example, Goldwyn *et al*. have empirically shown that an SDE model describing the biochemical kinetics of the channel better approximates the stochastic behaviour of the true discrete-state Markov chain model, compared with a Hodgkin–Huxley SDE formulation [20]. Taken together, these results motivated us to use stochastic reaction networks as a framework to describe the temporal evolution of the proportions of ion channels being in a set of possible configurations.

Given the increasing demand of stochastic models for describing ion channel gating, there is a need for inference approaches to estimate gating parameters. Despite many methods being available to estimate the parameters of deterministic models of ion channel gating [12,21–25], only a few have been developed for the stochastic case. For example, several methods have been proposed to estimate the total number of channels η from *macroscopic* voltage-clamp data (i.e. collected from whole-cell recordings with many channels) [26,27], while estimating the kinetics parameters. Estimating the total number of channels η in addition to the dynamic parameters can help identify the total maximal conductance g, when we model it as the product $g=g_{s}\eta$ between the single-channel conductance $g_{s}$ and the number of ion channels η. These existing methods are typically based on the assumption that the number of channels η follows a binomial distribution, while the dynamics of ion channel gating is described by deterministic models. However, it has been shown that classical estimators based on a binomial assumption of the number of open channels have some limitations and may result in misleading conclusions [28,29]. Besides, these methods might not be able to distinguish between noise due to stochastic gating and noise due to measurement errors in the data, as suggested by our simulation studies of §3*e*. Thus how to best infer the relationship between the noise observed in the ionic measurements and the total number of channels in the cell membrane contributing to gating is still unclear.

A first attempt was made by employing a Bayesian filter, *MacroR*, to calibrate the parameters of ligand-gated ion channel models to fit *macroscopic* voltage-clamp data [30]. The authors proved that their method provides better estimates than approaches that do not consider statistical dependence of successive measurements [31]. A similar approach was also employed by [32], who developed a method that outperformed both a classical Kalman filter [33] and a rate equation approach [34–36] when applied to patch-clamp data with realistic open-channel noise. Their framework also enables inclusion of orthogonal fluorescence data, increasing the level of identifiability of the unknown parameters, and the accuracy of their estimates. These methods can be extended to the case of voltage-gated ion channels. However, approaches that are based on filtering techniques are computationally expensive because an integration step of the differential moment equations (DMEs), and the corresponding updates in the correction step, are computed between every consecutive time points where the measurements are collected [37,38]. This complexity becomes extremely important in the case of long time series, like the ionic current traces obtained from voltage-clamp experiments, typically having a sampling frequency on the order of 10 kHz.

To overcome the limitations of the existing approaches, in this work we developed an inference procedure to estimate the parameters of stochastic models of voltage-gated channels to fit *macroscopic* voltage-clamp data, which avoids the computational complexity of the filtering approach by focusing on the time evolution of the first two moments of the stochastic process, rather than the process itself. To this end, we first write the stochastic model of ion channel gating in a state-space formulation, where the dynamic model consists of a stochastic reaction network describing ion channel gating. The measurement model links the measured ionic current traces to the ion channel open state configuration via an Ohmic equation [1]. This state-space formulation allows us to distinguish between measurement error and noise due to the number of channels η in the cell membrane that are contributing to stochastic gating. Finally, our proposed inference scheme is based on a maximum likelihood (ML) approach that uses the DMEs to describe the changes over time of the mean mm(t) and covariance PP(t) of the state $x\;x_{t}$ of the system, thus avoiding the well-known high computational complexity that characterizes filtering techniques [37,38], as we show in §3*g* with a direct comparison of our proposed method with a Kalman filter approach. After testing our method with simulation studies, including a comparison with standard methods, we apply it to estimate the parameters of a novel five-state ion channel model, to fit data recorded from whole-cell voltage-clamp experiments.

## 2. Methods

**Figure 2.**
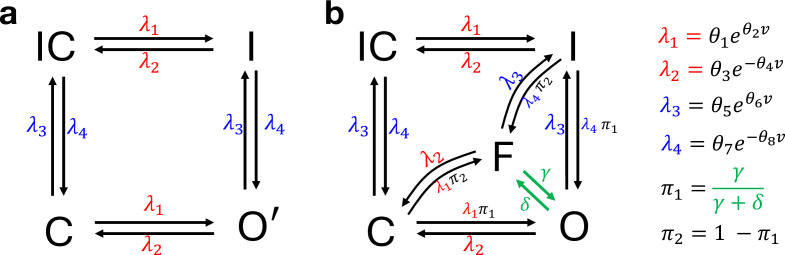
(a): Graphical representation of the four-state ion channel gating model from Beattie *et al.* [12] with the configurations: open conducting ${\text{O}}^{\prime }$, inactive I, closed C, and inactive-closed IC. (b): The five-state gating model used in this study and detailed in §2*f*. It extends the four-state model by splitting the open state ${\text{O}}^{\prime }$into open conducting O and open non-conducting (flickering) F states. In both models, arrows represent transitions between ion channel configurations whose rates are voltage-dependent, except for the flickering parameters γ and δ of the five-state model.

A graphical representation of our proposed framework is displayed in figure 1. Data consist of whole-cell patch-clamp recordings, including information on the total current yy flowing through the ion channels, observed over time under a particular voltage-clamp protocol $V_{\text{cmd}}$ which acts as a forcing function. The dynamics of channel gating is modelled with a stochastic reaction network formulation, describing the changes in channel configuration as a result of the variation in the cell membrane voltage. The possible channel configurations/states are unknown and cannot be measured, except for a linear transformation of the open probability, which is obtained via a measurement model based on an Ohmic formulation. Figure 2b shows the five-state ion channel model used in this study to analyse the experimental data. Inference is done via a maximum likelihood approach, that optimizes both dynamic and measurement parameters. The likelihood is based on the differential equations describing the first two moments mm and PP of the stochastic process xx, following a system of Itô-type SDEs. The system of SDEs is derived from the Fokker–Planck equation describing a channel’s reaction network. Our proposed statistical framework returns the optimal parameters describing the ion channel network, and the first two moments of the stochastic process xx and the current yy. This section includes the experimental design (§2*a*), our model formulation of ion channel gating based on stochastic reaction networks (§2*b*), its state-space formulation (§2*c*), a method to simulate synthetic data (§2*d*), the inference procedure (§2*e*), the definition of the five-state ion channel model (§2*f*), and a method used to compute box-constraints on the single-channel conductance as a function of the extracellular potassium concentration $[K{]}_{o}$ observed experimentally (§2*g*).

### (a) Experimental assay and voltage-clamp protocol

The data analysed in this work are collected from whole-cell voltage-clamp experiments performed on Chinese hamster ovary (CHO) cells overexpressing hERG1a (Kv11.1) [12]. Figure 1a shows an idealized model of a whole-cell voltage-clamp experiment, whereby a cell is placed into a solution, its membrane voltage $V_{\text{m}}$ is clamped (i.e. fixed) to a value $V_{\text{cmd}}$ with an electrically charged pipette, while the patch-clamp amplifier calculates, applies and reports the current $I_{out}$ necessary to maintain this voltage value across the cell membrane. In this ideal model, we assume that the command voltage $V_{\text{cmd}}$ equals the membrane voltage $V_{\text{m}}$, and so we refer simply to V below, while the measured current $I_{out}$ matches the total current flowing through the ion channels in the cell membrane. Some recent works do propose a relaxation of these assumptions which would introduce extra equations to account for patch-clamp artefacts [25,39,40]. After an experiment starts, the membrane voltage is clamped over time according to $V_{\text{cmd}}$ (figure 1b, top), inducing changes in the channels’ configurations (figure 1b, middle), and the total ionic current is observed (figure 1b, bottom). The current flowing through the channels is the only measurable quantity, whereas the channel configurations (e.g. open, closed, etc.) are unknown and cannot be measured directly.

**Figure 3.**
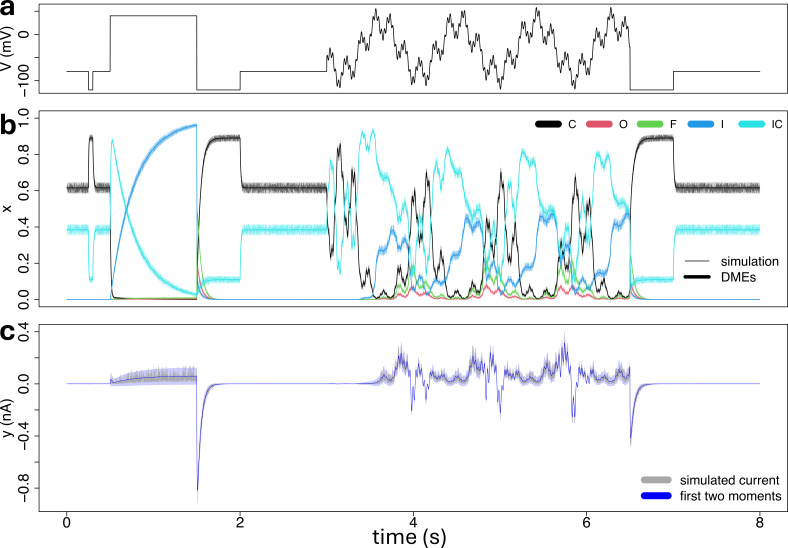
(a): The voltage-clamp protocol used in this study and detailed in §2*a*. It consists of the command voltage (*y*-axis) over time (*x*-axis). (b): Synthetic stochastic trajectories (thin lines) over time, for each ion channel configuration (colours), simulated from the five-state model by using the simulation scheme from §2*d*. The first two moments of the state vector xx are obtained from the differential moment equations (2.18), where the variance is displayed as a 95% confidence interval (shaded area) around the mean (line). The model parameters were set consistent with §3*a*, with a total number of channels equal to η=100. (c): Synthetic ionic current traces (grey) corresponding to the ion channel configurations of plot panel (b), and simulated from the measurement model of equation (2.11), with a measurement noise variance set to $\sigma^{2}=0\;{\text{nA}}^{2}$. The first two moments of the ionic current are obtained from equation (2.23), where the variance is displayed as a 95% confidence interval (blue-shaded area) around the mean (blue line).


Figure 3a displays the 8 s experimental voltage-clamp protocol, used in this work to characterize the current and train the stochastic ion channel models. This protocol features simple steps and a main sinusoidal section, defined as the sum of three sine waves of different amplitudes and frequencies. This protocol was originally designed to rapidly explore hERG channel kinetics with deterministic ion channel models [12,23]. The full protocol comprises a 250 ms phase at holding potential of −80 mV, followed by a 50 ms leak detection step to −120 mV, and then 200 ms back at −80 mV. This is followed by a 1 s activation step to 40 mV; a closing 500 ms step to −120 mV; and a return to −80 mV for 1 s. The 3.5 s sinusoidal portion of the protocol then follows, before a closing 500 ms step to −120 mV, and a return to −80 mV for 1 s. The main sinusoidal portion of the protocol is defined as

(2.1) $$V(t)=-30+\sum_{j=1}^{3}a_{j}\sin(\omega_{j}(t-t_{0}))\;,$$

where $a_{1}=54$ mV, $a_{2}=26$ mV, $a_{3}=10$ mV, $\omega_{1}=0.007{\text{ ms}}^{-1}$, $\omega_{2}=0.037{\text{ ms}}^{-1}$, $\omega_{3}=0.19{\text{ ms}}^{-1}$ and t is the time in milliseconds. Since the activation steps to +40 and −120 mV occur first, a time offset of $t_{0}=2500$ ms ensures the sine waves begin in phase. By design, this protocol allows us to explore both the physiological voltage- and time-dependence of hERG gating. The amplitudes $A_{j}$s of the sine waves were selected to keep the protocol within the physiological voltage range (−120,+60) mV; and the protocol frequencies $\omega_{j}$s were designed to investigate the characteristic physiological time scales of $I_{Kr}$ gating, ranging from 10 ms to 1 s [41,42]. More details on the protocol and experimental setup can be found in the original study [12].

### (b) A stochastic reaction model of ion channel gating

Consistent with our definition of a stochastic reaction network (electronic supplementary material, section A), we consider a Markov process


(2.2) $$x\;x_{t}=(x_{t1},\ldots ,x_{tn}{)}^{\prime }\;,\;0⩽x_{ti}⩽1\;,\;\sum_{i=1}^{n}x_{ti}=1\;,$$

describing the occupancy probabilities of n distinct ion channel configurations that evolve, in a time interval (t,t+Δt), according to a set of forward and backward biochemical reactions


(2.3) $$i\overset{\lambda_{ij}}{\to }j\;,\;j\overset{\lambda_{ji}}{\to }i\;,\;i\neq j\;,$$

where i and j are two different ion channel configurations. The corresponding reaction rates are defined as


(2.4) $$\lambda_{ij}=\theta_{1}\exp(\theta_{2}v_{t})\;,\;\lambda_{ji}=\theta_{3}\exp(-\theta_{4}v_{t})\;,$$

where $v_{t}$ is the value of the voltage protocol at time t, and $\theta_{j}$ (j=1,…,4) are positive scalar parameters. The negative sign inside the second reaction relates to the voltage-dependent terms being assumed proportional to the net charge movement along the trans-membrane electric field during the protein’s conformational change (i.e. movement of the imagined ‘charged particle’ in figure 1c up/down the electric field across the membrane for forward/backward reactions); see Jack *et al*. [43, p. 242].

Each reaction r=1,…,R


(2.5) $$r:l\overset{\lambda_{lm}}{\to }m\;,$$

describing the transition of ion channels from a configuration l to a configuration m with a rate $\lambda_{lm}$, has a net-effect vector $v\;v_{r}$ and a hazard function $h_{r}(x_{t},\theta )$ defined as


(2.6) $$v\;v_{r}=(\cdots \underset{l}{-1}\cdots \underset{m}{1}\cdots {)}^{\prime }\;,\;h_{r}(x\;x_{t},\theta \;\theta )=x_{tl}\lambda_{lm}\;,$$

where θθ is the vector of all the dynamic parameters involved in the system. Finally, we define the net-effect matrix and the hazard vector as


(2.7) $$\begin{array}{rl} V\;V & =\left[\begin{array}{ccc} v\;v_{1} & \cdots & v\;v_{R} \end{array}\right]\in \{-1,1{\}}^{n\times R}\;,\\ h\;h(x\;x_{t},\theta \;\theta ) & =(h_{1}(x\;x_{t},\theta \;\theta ),\ldots ,h_{R}(x\;x_{t},\theta \;\theta ){)}^{\prime }\in R^{R\times 1}\;, \end{array}$$

and we consider the Fokker–Planck approximation of the Master equation to obtain the Kolmogorov forward system (details in electronic supplementary material, sections B and C)


(2.8) $$\frac{\partial P(x\;x,t)}{\partial t}=-\nabla_{x\;x}\left\{\mu \;\mu (x\;x,\theta \;\theta )P(x\;x,t)\right\}+\frac{1}{2}\nabla_{x\;x}^{2}\left\{\beta \;\beta (x\;x,\theta \;\theta ,\eta )P(x\;x,t)\right\}\;,$$

as a probabilistic assumption for the stochastic process xx, where


(2.9) $$\begin{array}{r} \mu \;\mu (x\;x,\theta \;\theta )=V\;Vh\;h(x\;x,\theta \;\theta ),\;\beta \;\beta (x\;x,\theta \;\theta ,\eta )=\frac{1}{\eta }V\;V\left[\begin{array}{ccc} h_{1}(x\;x,\theta \;\theta ) & & \\ & \ddots & \\ & & h_{R}(x\;x,\theta \;\theta ) \end{array}\right]V\;V^{\prime }\; \end{array}$$

are the mean-drift and diffusion operators, and η is the total number of channels residing in the cell membrane.

### (c) State-space formulation of ion channel dynamics

Since the aim of this work is to estimate the parameters of the continuous-time stochastic model defined by equations (2.2)–(2.9) from whole-cell patch-clamp data that have been recorded at discrete-time points, we use a continuous-discrete state-space formulation that links the discrete-time measurements to the continuous-time stochastic process [44]. In particular, we consider a state-space formulation including a dynamic model defined as the system of Itô-type stochastic differential equations


(2.10) $$\begin{array}{c} \text{d}x=\mu (x,\theta )\text{d}t+\beta (x,\theta ,\eta )\text{d}W\;,\\ \text{d}W∼N_{n}(0,\text{d}tI_{n})\;, \end{array}$$

where μμ(xx,θθ) and ββ(xx,θθ,η) are defined by equation (2.9), coupled with a measurement model defined by the Ohmic equation


(2.11) $$\begin{array}{rl} y_{t} & =g_{s}\eta O(v_{t}-E)+\varepsilon \;,\\ y_{t} & =\underset{G_{t}}{\underbrace{g_{s}\eta (v_{t}-E)\left[\begin{array}{c} \overset{O}{0\cdots 0\;1\;0\cdots 0} \end{array}\right]}}x_{t}+\varepsilon \;,\;\varepsilon ∼N(0,\sigma^{2})\;, \end{array}$$

where $G\;G_{t}$ is a 1×n matrix having zero entries, except for the column(s) corresponding to the open conducting state(s), including the scaling factor


(2.12) $$g_{s}\eta (v_{t}-E)\;,$$

where $g_{s}$ is the single-channel conductance, η is the total number of channels, $v_{t}$ is the voltage at time t, E is the Nernst potential and $\sigma^{2}$ is the variance of the iid measurement error ε. Our proposed state-space formulation defined by equations (2.10) and (2.11) can be interpreted as a hidden Markov model where all the configurations in the state vector xx are latent, and only the occupancy probability for the open state O, scaled by the Ohmic term (2.12), can be observed through the measurement model of equation (2.11).

### (d) Simulating stochastic reaction networks of ion channel gating

Here we describe our proposed method to simulate trajectories that are solutions of equation (2.10), having a mean drift and diffusion operators consistent with equation (2.9). The solutions $\{x\;x_{t}{\}}_{t}$ of this system are n-dimensional vectors describing the time-evolution in the proportion of channels that are in the n configurations, and therefore must remain non-negative and sum to 1, in order to be biologically meaningful. To this end, instead of simulating the trajectories of the original process $\{x\;x_{t}{\}}_{t}$, we use the Euler–Maruyama method [45] to simulate the trajectories of the transformed process


(2.13) $$\begin{array}{rl} \xi \;\xi_{t} & ={\left(\xi_{t1},\ldots ,\xi_{tn}\right)}^{\prime }\;,\\ \xi_{ti} & =g(x_{ti})=\log\frac{x_{ti}}{1-x_{ti}}\;,\;i=1,\ldots ,n\;, \end{array}$$

which is obtained by applying the Itô lemma D.1 from electronic supplementary material, section D, to each component of the process $x\;x_{t}$ and the transformation g. Then, the original process is obtained via the inverse transform of g as


(2.14) $$\begin{array}{rl} x\;x_{t} & ={\left(x_{t1},\ldots ,x_{tn}\right)}^{\prime }\;,\\ x_{ti} & =g^{-1}(\xi_{ti})=\frac{1}{1+\exp(\xi_{ti})}\;,\;i=1,\ldots ,n\;. \end{array}$$

The inverse transform $g^{-1}$ ensures that $x_{ti}\in (0,1),\;i=1,\ldots ,n$, and, therefore, our proposed simulation approach does not need any special reflection boundaries like those proposed by [46], and the original set of SDEs describing the biochemical formulation of ion channel gating is preserved. Finally, to make sure the states sum to 1, we simulate the trajectories of the first n−1 states, and the last one is defined as


(2.15) $$x_{tn}=1-\sum_{i=1}^{n-1}x_{ti}\;,$$

for each time t. The pseudo-code of the Euler–Maruyama simulation scheme is reported in algorithm 1 of electronic supplementary material, Section D1.

### (e) Inference procedure

Consider the state-space model defined by equations (2.10) and (2.11). Let $y\;y_{1:\tau }$ be the vector of measurements collected at times $t=t_{1},\ldots ,t_{\tau }$, and $x\;x_{1:k}$ the process states from time $t_{1}$ up to $t_{k}$, where k=1,…,τ. Assuming that the Markov properties from electronic supplementary material, section E, hold for the distributions involved in the dynamic and measurement models of equations (2.10) and (2.11), then, consistent with electronic supplementary material, section F, the first two moments


(2.16) mm=E[xx],PP=V(xx),

of a solution xx to the system of Itô-type SDEs (2.10), and their partial derivatives,


(2.17) $$\frac{\partial m\;m}{\partial \psi_{j}}\;,\;\frac{\partial P\;P}{\partial \psi_{j}}\;,$$

follow the differential moment equations (DMEs) defined as


(2.18) $$\begin{array}{rlrl} \frac{\text{d}m\;m}{\text{d}t} & =J\;J_{\mu \;\mu ,x\;x}m\;m\;, & m\;m(0) & =m\;m_{\infty }\;,\\ \frac{\text{d}P\;P}{\text{d}t} & =J\;J_{\mu \;\mu ,x\;x}P\;P+P\;PJ\;J_{\mu \;\mu ,x\;x}^{\prime }+\beta \;\beta (m\;m,\theta \;\theta ,\eta )\;, & P\;P(0) & =P\;P_{\infty }\;, \end{array}$$

coupled with their sensitivities defined as


(2.19) $$\begin{array}{rlrl} \frac{\text{d}}{\text{d}t}\frac{\partial m\;m}{\partial \psi_{j}} & =\frac{\text{d}}{\text{d}\psi_{j}}\left(J\;J_{\mu \;\mu ,x\;x}m\;m\right)\;, & \frac{\partial m\;m}{\partial \psi_{j}}(0) & =\frac{\partial m\;m_{\infty }}{\partial \psi_{j}}\;,\\ \frac{\text{d}}{\text{d}\psi_{j}}\frac{\partial P\;P}{\partial \psi_{j}} & =\frac{\text{d}}{\text{d}\psi_{j}}\left(J\;J_{\mu \;\mu ,x\;x}P\;P+P\;PJ\;J_{\mu \;\mu ,x\;x}^{\prime }+\beta \;\beta (m\;m,\theta \;\theta ,\eta )\right)\;, & \frac{\partial P\;P}{\partial \psi_{j}}(0) & =\frac{\partial P\;P_{\infty }}{\partial \psi_{j}}\;, \end{array}$$

where μμ(xx,θθ) and ββ(xx,θθ,η) are defined by equation (2.9), $J\;J_{\mu \;\mu ,x\;x}$ is the Jacobian matrix of μμ(xx,θθ) with respect to xx and evaluated at mm, and


(2.20) $$\psi \;\psi =(\theta \;\theta ,\eta ,g_{s},\sigma^{2}{)}^{\prime }$$

is the vector of all unknown parameters (see electronic supplementary material, equation G3). Furthermore, due to the patch-clamp experimental conditions, we know that the system is at steady state at t=0, and therefore $m\;m_{\infty }$ and $P\;P_{\infty }$ are defined as the state vectors satisfying the steady conditions


(2.21) $$\frac{\text{d}m\;m}{\text{d}t}=0\;0\;,\;\frac{\text{d}P}{\text{d}t}=0\;0\;,$$

which, in our case, have an explicit formulation. Furthermore, by applying the properties of linear transformation of a multivariate Gaussian distribution to the measurement model of equation (2.11), the log-likelihood is given by


(2.22) $$\ell (\psi \;\psi |y\;y_{1:\tau })=-\sum_{k=1}^{\tau }\log s_{k}-\sum_{k=1}^{\tau }\frac{1}{s_{k}}(y_{k}-{\hat{y}}_{k}{)}^{2}\;,$$

where


(2.23) $$\begin{array}{rl} {\hat{y}}_{k} & =Og_{s}\eta (v_{k}-E)=G\;G_{k}m\;m_{k}\;,\\ s_{k} & =G\;G_{k}P\;P_{k}G\;G_{k}^{\prime }+\sigma^{2}\;. \end{array}$$

The gradient $\nabla_{\psi \;\psi }\ell (\psi \;\psi |y\;y_{1:\tau })$ of the log-likelihood with respect to each component of ψψ is reported in electronic supplementary material, section H. The transformed vector parameter


(2.24) $$\phi \;\phi =(\log\theta \;\theta ,\log(\eta -1),\log g_{s},\log\sigma^{2}{)}^{\prime }$$

of the log-likelihood is estimated by solving the optimization problem


(2.25) $$\hat{\phi \;\phi }=\underset{\phi \;\phi }{\text{argmin}}-\ell (\phi \;\phi |y\;y_{1:\tau })\;,$$

by using the L-BFGS algorithm, to which we provide the log-likelihood and its gradient. At each step of the optimization, to evaluate the log-likelihood $\ell (\psi \;\psi |y\;y_{1:\tau })$, and its gradient $\nabla_{\psi \;\psi }\ell (\psi \;\psi |y\;y_{1:\tau })$, we numerically solve the differential moment equations (2.18), and their sensitivities defined by equation (2.19), using the method from electronic supplementary material, section G.

### (f) A 5-state model of ion channel gating

We extended the 4-state ion channel model of hERG gating proposed by Beattie *et al*. [12], displayed in figure 2a, by following Bett *et al.* [47] in introducing an additional Markov state F describing a non-conducting ‘flickering’ open configuration, leading to the 5-state model which is displayed in figure 2b. This choice is motivated by the experimental results suggesting that after depolarization, single channels open, but ‘*flicker*’ rapidly between conducting O and non-conducting F states [48]. The flickering provides a rapid opening and closing, that does not depend on the voltage-dependent processes of activation or inactivation, but may represent unrelated physical changes such as fluctuations in the selectivity filter or blocking by divalent ions, and can account for the apparent contradictions between macroscopic data and single channel measurements [47].

The voltage-dependent reaction rates are defined as


(2.26) $$\begin{array}{rlrl} \lambda_{1} & =\theta_{1}e^{\theta_{2}v}\;, & \lambda_{3} & =\theta_{5}e^{\theta_{6}v}\;,\\ \lambda_{2} & =\theta_{3}e^{-\theta_{4}v}\;, & \lambda_{4} & =\theta_{7}e^{-\theta_{8}v}\;, \end{array}$$

as per equation (2.4). The new flickering mechanism is described by the voltage-independent reaction rates γ and δ between the O and F states. The values of the flickering reaction rates are fixed to γ=1/6.7 ms and δ=1/2.5 ms, corresponding to the open and intermediate closed dwell times measured experimentally at +100 mV [48]. Finally, to preserve microscopic reversibility, the reactions that bring an ion channel configuration into either the open conducting state O, or the flicker (open non-conducting) state F are scaled, respectively, by


(2.27) $$\pi_{1}=\frac{\gamma }{\gamma +\delta }\;,\;\pi_{2}=1-\pi_{1}\;.$$

Thus, our proposed five-state model is obtained from the four-state model [12], after splitting the open configuration $O^{\prime }$ into the open conducting O and flickering non-conducting F states.

### (g) Constraints on single-channel conductance

Here, we detail a method to construct box-constraints, i.e. upper and lower bounds, on the single-channel conductance $g_{s}$, as a function of the experimentally observed extracellular potassium concentration $[K{]}_{o}$. A work on hERG cRNA injected into Xenopus oocytes, with currents measured from single channels, suggests that single-channel conductance $g_{s}$ depends on the extracellular potassium concentration $[K{]}_{o}$ [49]. The authors observed a single-channel conductance of 7.0, 10.1 and 13.7 pS at 50, 100 and 300 mM $[K{]}_{o}$, respectively. Consistent with the original study, we fitted a Michaelis–Menten function


(2.28) $$\log\;g_{s}([K{]}_{\text{o}})=\log\left(\frac{g_{s,max}}{1+\frac{[K{]}_{\text{50}\%}}{[K{]}_{\text{o}}}}\right)\;,$$

to the data, where $g_{s,max}$ and $[K{]}_{\text{50\%}}$ are the single-channel conductance saturation level and the extracellular potassium concentration at half maximal conductance, respectively. The log⁡ function in equation (2.28) ensures that the response (a conductance) is always positive. These parameters are unknown and have to be inferred from the data. The parameters $g_{s,max}$ and $[K{]}_{\text{50\%}}$ are inferred from the single-channel/potassium concentration data of Kiehn *et al.* [49], by using a nonlinear least-squares approach, where the sum of squared residuals between data and predictions, given by the model of equation (2.28), is minimized. Then, the box-constraints for the single-channel conductance $g_{s}$ are defined as the 1−α confidence interval with lower and upper bounds given by


(2.29) $$g_{\text{s},\text{lb}}=\exp\left(\log{\hat{g}}_{\text{s}}([K{]}_{\text{obs}})-z_{1-\alpha /2}\hat{\tau }\right)\;,\;g_{\text{s},\text{ub}}=\exp\left(\log{\hat{g}}_{\text{s}}([K{]}_{\text{obs}})+z_{1-\alpha /2}\hat{\tau }\right)\;,$$

where $[K{]}_{\text{obs}}$ is the extracellular potassium concentration observed experimentally (4 mM in our case), ${\hat{g}}_{\text{s}}([K{]}_{\text{obs}})$ is the corresponding model prediction, $z_{1-\alpha /2}$ is the (1−α/2)-quantile of a standard Gaussian distribution, with level set to α=0.01, and ${\hat{\tau }}^{2}$ is the corrected sample variance estimator. Parameter estimates, model fit, and the box-constraints inferred from the single-channel/potassium concentration data of Kiehn *et al.* [49] are reported in §3*i*. This method is used in §3*i*, before fitting the five-state model to the whole-cell voltage-clamp data, to make sure that the single-channel conductance $g_{\text{s}}$ takes only physiologically plausible/sensible values, consistent with the experimental findings of [49].

## 3. Results

We tested our proposed inference method in several simulation studies in terms of: (i) uncertainty quantification for the inferred parameters, (ii) ability to distinguish between measurement error and stochastic noise, including a comparison with a previously published method, (iii) scalability to complex gating scenarios, (iv) computational complexity, including a direct comparison with a Kalman filter approach, and (v) misspecification of the flickering mechanism. After validating our method, we applied it to analyse whole-cell voltage-clamp data collected from nine CHO cells under a recently designed sinusoidal voltage-clamp protocol [12]. The details and results of the specific analyses are reported in the next subsections.

### (a) Simulation setting

In each simulation either the five-state ion channel reaction network of §2*f* or the eight-state ion channel model of §3*f* has been used as the true data-generating process, depending on the particular goal. Each model is written as the stochastic reaction network formulation of equations (2.2)–(2.9) from §2*b*, and its Itô-type formulation of equation (2.10). For the five-state model, the voltage-dependent reaction rates of equation (2.4) reduce to those defined by equation (2.26) from §2*f*. The 8-dimensional dynamic vector parameter


(3.1) $$\theta \;\theta =(\theta_{1},\theta_{2},\theta_{3},\theta_{4},\theta_{5},\theta_{6},\theta_{7},\theta_{8}{)}^{\prime }\;,$$

for the five-state model, has the values reported in table 1, whereas for the 16-dimensional dynamic vector parameter of the eight-state model we used the values reported in table 2. For both the five- and eight-state ion channel models, the reaction rates of the flickering mechanism were set to γ=1/6.7 ms and δ=1/2.5 ms, and the rates of the reactions that bring an ion channel configuration into either the open conducting state *O* or the flicker state *F* were scaled, respectively, by $\pi_{1}$ and $\pi_{2}$, according to equation (2.27) of §2*f*. Then, we used the Euler–Maruyama simulation scheme from §2*d* to generate synthetic stochastic traces of the ion channel configurations, combined with the measurement model of equation (2.11) to obtain the corresponding synthetic ionic current traces. For the measurement model of equation (2.11), the total cell conductance

**Table 1.** Values of the 8-dimensional dynamic vector parameter θθ, and the whole cell hERG conductance g , used in equations (2.10) and (2.11) to generate the synthetic stochastic traces from the five-state model of §2*f* for the validation studies.

$\theta_{1}$	$\theta_{2}$	$\theta_{3}$	$\theta_{4}$	$\theta_{5}$	$\theta_{6}$	$\theta_{7}$	$\theta_{8}$	g
2.23e−4 ${\text{ms}}^{-1}$	7.01e−2 ${\text{mV}}^{-1}$	3.41e−5 ${\text{ms}}^{-1}$	5.45e−2 ${\text{mV}}^{-1}$	8.71e−2 ${\text{ms}}^{-1}$	8.26e−3 ${\text{mV}}^{-1}$	5.40e−3 ${\text{ms}}^{-1}$	3.24e−2 ${\text{mV}}^{-1}$	1.46e−1 μS

**Table 2.** Values of the 16-dimensional dynamic vector parameter θθ , the whole cell hERG conductance g , and the measurement error variance $\sigma^{2}$ , used in equations (2.10) and (2.11) to generate the synthetic stochastic traces from the eight-state model of §3*f* for the validation studies.

$\theta_{1}$	$\theta_{2}$	$\theta_{3}$	$\theta_{4}$	$\theta_{5}$	$\theta_{6}$	$\theta_{7}$	$\theta_{8}$	$\theta_{9}$
1.11e−1 ${\text{ms}}^{-1}$	1.69e−3 ${\text{mV}}^{-1}$	4.61e−3 ${\text{ms}}^{-1}$	2.01e−2 ${\text{mV}}^{-1}$	1.04e−1 ${\text{ms}}^{-1}$	9.70e−3 ${\text{mV}}^{-1}$	5.27e−3 ${\text{ms}}^{-1}$	3.12e−2 ${\text{mV}}^{-1}$	2.61e−2 ${\text{ms}}^{-1}$

$\theta_{10}$	$\theta_{11}$	$\theta_{12}$	$\theta_{13}$	$\theta_{14}$	$\theta_{15}$	$\theta_{16}$	g	$\sigma^{2}$
2.08e−3 ${\text{mV}}^{-1}$	5.74e−4 ${\text{ms}}^{-1}$	4.34e−2 ${\text{mV}}^{-1}$	3.10e−4 ${\text{ms}}^{-1}$	6.55e−2 ${\text{mV}}^{-1}$	2.63e−2 ${\text{ms}}^{-1}$	3.55e−2 ${\text{mV}}^{-1}$	1.73e−1 μS	1.00e−5 ${\text{nA}}^{2}$


(3.2) $$g=g_{s}\eta \;,$$

was fixed to the values reported in tables 1 and 2 for the five- and eight-state models respectively, in each simulation study, whereas η and the measurement noise variance $\sigma^{2}$ are tuned across the different simulation studies, so as to mimic a diverse set of scenarios of stochastic noise and measurement error. The true single-channel conductance $g_{s}$ is then derived from equation (3.2). For the simulations, in both the dynamic model and the measurement model, the input v for the voltage was set to the sinusoidal voltage-clamp protocol developed by [12] and reported in figure 3a. A detailed description of the sinusoidal voltage protocol can be found in §2*a*, and in the original study [12]. Figure 3b,c shows an example of a synthetic trace of ion channel configurations, together with the corresponding current trace, that have been generated from the five-state ion channel reaction network of figure 2b, under this setting, by using our proposed simulation scheme of §2*d*, where the number of channels and the measurement error noise variance were set to η=100 and $\sigma^{2}=0$ ${\text{nA}}^{2}$, respectively. For a comparison, in figure 3b, we also display the first two moments of the process obtained by using the differential moment equations (2.18), whereas in figure 3c, we report the first two moments of the ionic current, by using equation (2.23). In both cases, the second moment is displayed as a 95% confidence interval around the mean trace.

### (b) Uncertainty quantification across independent stochastic traces

We used the simulation scheme of §2*d* to generate N=20 synthetic current traces of η channels from the true generative data process of figure 2b, under the simulation setting of §3*a*. Synthetic data were generated under η=100, 1000 or 10000; and measurement error variance $\sigma^{2}=10^{-6}$, $10^{-5}$ or $10^{-4}$ ${\text{nA}}^{2}$; to mimic a diverse set of scenarios for stochastic noise versus measurement error. The values for the dynamic parameters θθ, the total membrane conductance g and the rates γ and δ of the flickering mechanism used for the simulations are reported in §3*a*. Then we used our proposed inference method of §2*e* to recover all the parameters, independently from each generated synthetic trace. During inference, all the parameters are assumed to be unconstrained.

**Figure 4.**
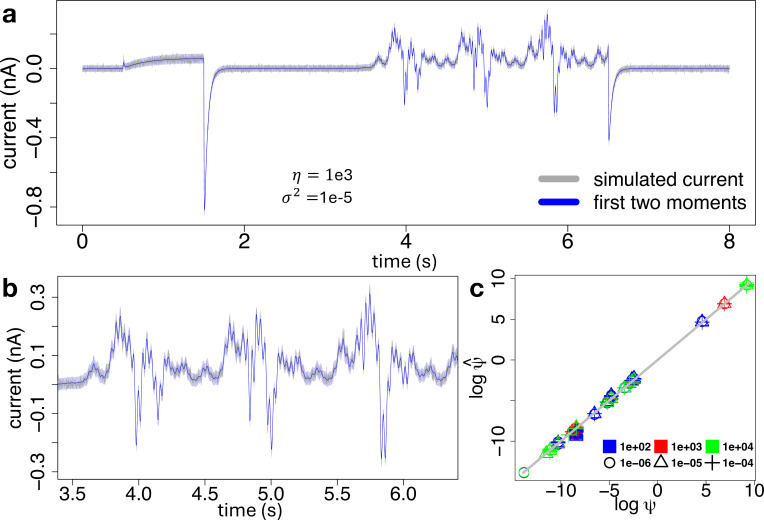
(a): A synthetic current trace (grey) generated under η=1000 and $\sigma^{2}=10^{-5}$ ${\text{nA}}^{2}$, along with the first two moments of the model fit, defined by equation (2.23) and obtained with our proposed inference method from §2*e*, where the variance is displayed as a 95% confidence interval (blue-shaded area) around the mean (blue line). (b): A zoom of plot panel (a) on a portion of the sinusoidal part of the voltage-clamp protocol. (c): Scatter plot of the estimated against true parameters ψψ, on a log–log scale, under different combinations of total number of channels η (colours) and variance $\sigma^{2}$ of the measurement error (symbols). Each replicate correspond to a particular synthetic trace. The diagonal grey line identifies the true values.

Results are displayed in figure 4, suggesting that our inference method is able to recover the true parameters under any combination of measurement error and stochastic noise. The last two can be distinguished by our method, as can be seen from the bottom-left and top-right corners of figure 4c, where the parameter estimates always lie close to the diagonal grey line. Furthermore, our proposed inference method is robust across the independent stochastic traces. Indeed, for each combination of measurement error and stochastic noise the corresponding N=20 independent parameter estimates show low variation around the true values identified by the grey diagonal line.

### (c) Practical parameter identifiability

Structural non-identifiability is the result of functionally related model parameters, where it is impossible for given model output(s) (e.g. in this context, recording only current) to give independent information on all the model parameters no matter what experiment is performed [50]. Global sensitivity analysis could also be used to explore structural non-identifiability of mathematical models [51]. However, even with structural identifiability in place, practical non-identifiability can still arise when we have poor parameter estimates due to limited amounts or quality of data [52].

More formally, given a log-likelihood ℓ(ψψ|yy), the jth component $\psi_{j}$ of ψψ is structurally non-identifiable if its variation can be completely compensated by calibrating the remaining parameters $\psi \;\psi_{i\neq j}$, thus having no impact on the likelihood. This means that the data yy cannot provide any information about $\psi_{j}$. However, if $\psi_{j}$ is structurally identifiable but there is an high level of uncertainty on the corresponding estimate ${\hat{\psi }}_{j}$ due to limited amount and quality of experimental data yy, then we will have a high degree of uncertainty in our estimate of $\psi_{j}$ and it is said to be practically non-identifiable [52]. These scenarios of non-identifiability are well reflected by the shape of the profile likelihood, as described in the electronic supplementary material, section J. Here, we assess practical identifiability by attempting to (re)fit parameters that we used to generate some data, noting that good practical identifiability guarantees (at least locally) structural identifiability.

Using the five-state model, we have performed an additional simulation study, based on the profile likelihood approach, in order to check practical identifiability of the vector parameter ψψ defined by equation (2.20), characterizing the log-likelihood of equation (2.22). In particular, we used the simulation scheme of §2*d* to generate one synthetic current trace of η channels from the true generative data process of figure 2b, under the simulation setting of §3*a*. Synthetic data were generated under η=1000 and $\sigma^{2}=10^{-5}{\text{nA}}^{2}$. The values for the dynamic parameters θθ, the total membrane conductance g, and the rates γ and δ of the flickering mechanism used for the simulations are listed in §3*a*.

**Figure 5.**
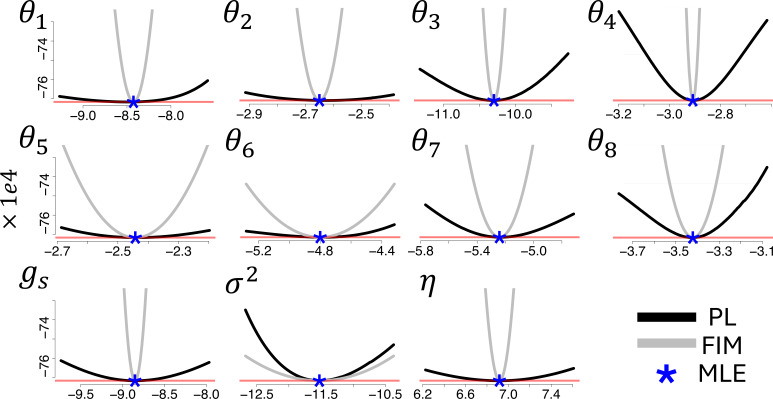
Profile negative log-likelihood (PL, black line) and Fisher Information Matrix-based parabolas (FIM, grey lines) evaluated for the logarithm of each parameter (plot panels). The ML estimates are indicated by blue star symbols. All profile negative log-likelihoods are displayed on the same scale for the y-axis, so as to compare their level of identifiability. The red horizontal lines identify the threshold $-l(\hat{\psi \;\psi })+1/2\chi_{1-\alpha ,1}^{2}$, representing a 95% confidence interval.


We applied the profile likelihood approach [52], detailed in electronic supplementary material, section J, to the simulated data. To build the profile likelihood defined in electronic supplementary material, equation (J1), each parameter $\psi_{j}$ was varied by ±10% from the absolute value of its maximum likelihood estimator ${\hat{\psi }}_{j}$, whereas the remaining nuisance parameters $\psi \;\psi_{i\neq j}$ were optimized by using our proposed inference scheme from §2*e*. During inference, all the parameters are assumed to be unconstrained. Results are displayed in figure 5, suggesting that each parameter is identifiable, as their profile negative log-likelihoods are not flat and increase when the parameter of interest deviates from its ML estimator. In particular, each profile negative log-likelihood rapidly exceeds the threshold,

(3.3) $$-l(\hat{\psi \;\psi })+1/2\chi_{1-\alpha ,1}^{2}\;,$$

defining a (1−α) confidence interval for ψψ, displayed as red horizontal lines in figure 5 for α=0.05, where $\hat{\psi \;\psi }$ is the maximum likelihood estimator of ψψ (see electronic supplementary material, section J, for details).

Similar findings are obtained with Fisher Information-based parabolas, which we computed for each parameter according to electronic supplementary material, section I and displayed in figure 5, together with the profile negative log-likelihood. The comparison of the results obtained by both methods suggests that the peaked FIM-based parabolas makes the profile likelihood approach more parsimonious in evaluating the identifiability of the model parameters, as the confidence intervals defined by the profile negative log-likelihoods are always wider than the FIM counterparts at any level α, except for the measurement noise parameter $\sigma^{2}$.

Furthermore, in order to make sure that our inference method, based on the local optimizer L-BFGS, is able to recover the profile likelihood accurately, we empirically show that the log-likelihood defined by equation (2.22) is locally well approximated by a quadratic function of the parameters in the interval defined as ±10% the absolute values of the maximum likelihood estimator around $\hat{\psi \;\psi }$, that is


(3.4) $$I_{\hat{\psi \;\psi },\pm 10\%}=[\hat{\psi \;\psi }-|\hat{\psi \;\psi }|/10,\hat{\psi \;\psi }+|\hat{\psi \;\psi }|/10]\;,$$

the range over which we estimated the profile likelihood in figure 5. To this end, we applied our inference method to estimate the parameters from the same simulated trace previously used to estimate the profile likelihood, and the inference is repeated by using N=100 different starting guesses for the vector parameter ψψ, uniformly sampled in $I_{\hat{\psi \;\psi },\pm 10\%}$. Inference results are displayed in electronic supplementary material, figure S1, showing that the parameter estimates obtained by our method from each starting guess are close to the true values. This result suggests that the estimated values of the profile likelihood from figure 5 are accurate, at least regionally in the interval $I_{\hat{\psi \;\psi },\pm 10\%}$. Note that the parameter region defined by equation (3.4), where the log-likelihood behaves as a quadratic function of the parameters, largely covers the (1−α) confidence interval for ψψ, with α=0.05, as displayed in figure 5. However, to explore possible multimodal behaviours that might arise beyond the region $I_{\hat{\psi \;\psi },\pm 10\%}$, our inference method can be easily extended by replacing the local optimizer L-BFGS with a global counterpart.

### (d) Uncertainty due to sampling frequency

We assessed our proposed inference method in terms of robustness against parameter uncertainty due to sampling ω, defined as the fraction of data observed out of the complete set of measurements. To generate the stochastic traces, consistent with §3*b*, we first used the simulation scheme of §2*d* to generate N=100 independent synthetic current traces of η channels from the five-state model under the simulation setting of §3*a*. Synthetic data were generated under η=1000 total number of ion channels, with a variance for the measurement error set to $\sigma^{2}=10^{-5}{\text{ nA}}^{2}$. Subsequently, a sampling ω∈{90,80,70,60,50,40,30,20,15,10,5}% has been applied to each synthetic trace to obtain reduced traces of various levels of information from the entire trace. The sampling process has been applied uniformly across time.

**Figure 6.**
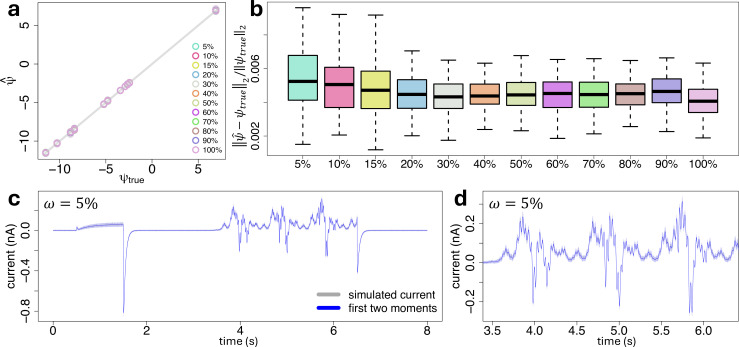
(a): Scatter plot of estimated (y-axis) against true (x-axis) parameters, on a log-log scale, under each level of sampling frequency ω (colours), with a total number of channels η=1000 and measurement error variance $\sigma^{2}=10^{-5}{\text{nA}}^{2}$. The corresponding relative error between the estimated and true parameters are displayed in panel (b), where $\|\cdot {\|}_{2}$ is the Euclidean norm. (c): One synthetic current trace obtained from the full trace with a sampling frequency of ω=5% (grey), and the corresponding model fit, obtained with our inference method, in terms of the first two moments defined by equation (2.23), where the variance is displayed as a 95% confidence interval (blue-shaded area) around the mean (blue line). Model fits under different values of sampling frequencies are displayed in electronic supplementary material, figure S2. (d): A zoom of plot panel (c) on a portion of the sinusoidal part of the voltage-clamp protocol.

We then used our proposed inference method of §2*e* to recover all the parameters, independently from each generated synthetic trace under each level of sampling ω. During inference, all the parameters are assumed to be unconstrained. Results are displayed in figure 6, suggesting that our inference method is able to recover the true parameters under any level of sampling frequency ω, even for the extreme scenarios where we only analyse ω=5% of the entire trace, as shown by figure 6a. Consistent with §3*b*, our proposed inference method is robust across the independent stochastic traces, as shown by the low variation of the N=100 independent parameter estimates around the true values identified by the grey diagonal line, for each level of sampling ω. In particular, the relative error between the estimated and true parameters only increases slightly as the sampling ω decreases, as suggested by the box plots of figure 6b, confirming that our proposed inference method is robust with any source of uncertainty given by unobserved measurements due to experimental sampling limits. Results are confirmed in terms of model fit, for which we show in figure 6c,d, the predictions of the first two moments of a single synthetic current trace obtained from the full trace with a sampling of ω=5%. The model fits for the entire trace (ω=100%) and under different values of sampling frequencies ω are displayed in figure 4a and electronic supplementary material, figure S2, respectively.

### (e) Number of channels and measurement noise

With an additional simulation study, we checked whether our proposed method is able to recover the true total number of channels (η) and the measurement error variance ($\sigma^{2}$), and to distinguish between the corresponding sources of noise. In this regard, we compared the performance of our method with the standard approach [27]. As per §3*b*, we used the simulation scheme of §2*d* to generate N=100 ionic current traces following the five-state model under the simulation setting of §3*a*. Each synthetic current trace was simulated with a different value of η, ranging in the interval $\left[10^{2},10^{4}\right]$. The values for the dynamic parameters θθ, the total membrane conductance g, and the rates γ and δ of the flickering mechanism used in the state-space model of equations (2.10) and (2.11) for the simulations are listed in §3*a*. The simulations have been repeated under three different values of the measurement noise variance $\sigma^{2}$ $\left(10^{-6},10^{-5},10^{-4}{\text{nA}}^{2}\right)$. Consistent with the state-space model formulation of equations (2.10) and (2.11), this simulation study has been designed so that an increase in η leads to a decrease in the stochastic noise, whereas an increase in $\sigma^{2}$ leads to an increase in the measurement error.

**Figure 7.**
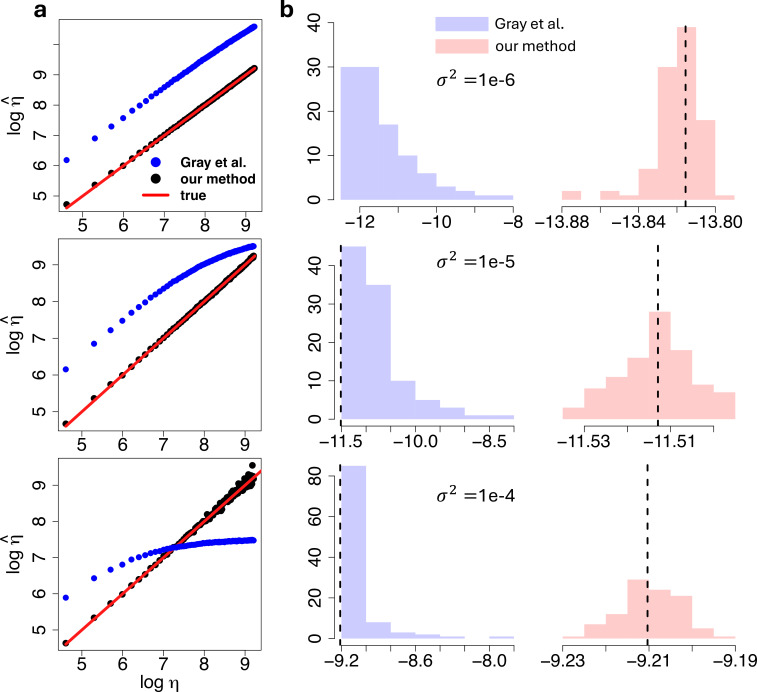
(a): Scatter plot of the estimated against true number of channels η, on a log-log scale, obtained by our proposed method (black) and the standard approach (blue). The 45° diagonal red line indicates the true values. (b): Histogram of the estimated measurement noise variance $\sigma^{2}$, on a log scale, provided by either our proposed method (red) and the standard approach (blue). Results displayed in both left and right panels are obtained from the analyses of the synthetic ionic current traces generated under a measurement noise variance $\sigma^{2}$ set to $10^{-6}$ (top), $10^{-5}$ (middle) and $10^{-4}$ ${\text{nA}}^{2}$ (bottom).

We then applied our proposed inference method from §2*e* and the competitor method [27] on the simulated data. During inference all the parameters are assumed to be unconstrained. Results are reported in figure 7, suggesting that our proposed method is able to recover both the true number of channels η and the measurement noise variance $\sigma^{2}$ in each case. Indeed the scatter plots of figure 7a show that the estimates of η provided by our method overlap the diagonal red line, which identifies the true values. Whereas, the number of channels inferred by the standard method [27] deviates consistently from the true values, as suggested by the parameter estimates. Furthermore, the histograms of figure 7b suggest that our method also outperforms this existing approach in estimating $\sigma^{2}$, and that our method better distinguishes between the sources of stochastic noise and measurement error.

### (f) Scalability to more complex gating

**Figure 8.**
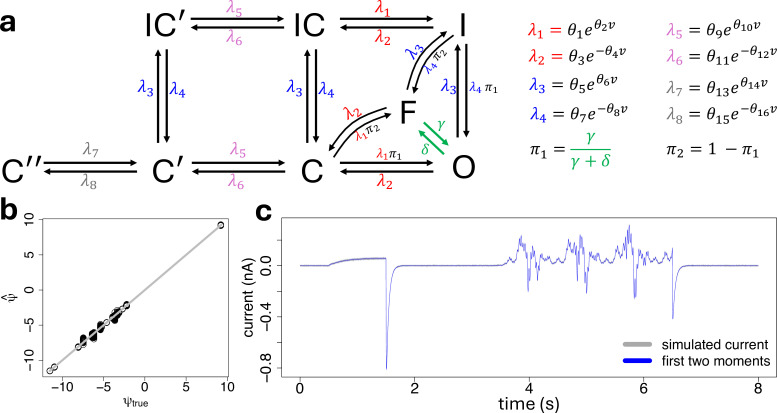
(a): Graphical representation (left) and reaction rates (right) of the eight-state ion channel gating model used in this study. It extends the five-state model of figure 2b by splitting the closed configuration into three separate states C, ${\text{C}}^{\prime }$, ${\text{C}}^{\prime \prime }$, and the inactive-closed configuration into two separate states IC and ${\text{IC}}^{\prime }$. (b): Scatter plot of estimated (y-axis) against true (x-axis) parameters, on a log–log scale, obtained by applying our inference method of §2*e* to calibrate the eight-state ion channel gating model of plot panel (a) to fit the N=100 synthetic traces, independently. (c): One synthetic stochastic current trace (grey) simulated with the Euler–Maruyama electronic supplementary algorithm 1 from the eight-state ion channel gating network of plot panel (a) with the dynamic parameters set to the values reported in table 2 and a total number of ion channels set to η=10000, together with the corresponding model fit, in terms of the first two moments defined by equation (2.23), where the variance is displayed as a 95% confidence interval (blue-shaded area) around the mean (blue line).

We assessed our proposed inference method under modelling scenarios that are more challenging compared with the five-state model discussed above. To this end we considered the eight-state ion channel gating model of figure 8a, which we coded in the state-space model formulation of equations (2.10) and (2.11) using the stochastic reaction network formulation of equations (2.2)–(2.7) and the Fokker–Planck approximation of equations (2.8) and (2.9). Effectively, the eight-state model extends the five-state model by splitting the closed configuration into three separate states C, ${\text{C}}^{\prime }$, ${\text{C}}^{\prime \prime }$, and by splitting the inactive-closed configuration into two separate states IC and ${\text{IC}}^{\prime }$. This extended configuration led to a system of 7 ODEs describing the changes in time of the mean mm of the process xx, 28 ODEs describing the changes in time of the lower-diagonal part of the covariance matrix PP of the process xx, and 560 sensitivity ODEs describing the changes in time of the partial derivatives of the differential moment equations (2.18) with respect to the dynamic parameters. This gives a complete system of 595 ODEs having a dimension much higher than the full system of 126 ODEs characterizing the simpler five-state model. Therefore, we consider this exercise a good benchmark to assess the scalability of our method for calibrating more complex gating models.

We simulated N=100 independent stochastic traces from the eight-state model, under the sinusoidal voltage protocol, by using the Euler–Maruyama algorithm 1 of electronic supplementary material, section D1. The values for the dynamic parameters θθ, the total membrane conductance g and the rates γ and δ of the flickering mechanism used in the state-space model of equations (2.10) and (2.11) for the simulations are reported in table 2, and we set the total number of ion channels to η=10000. We then applied our proposed inference method from §2*e* on the simulated data. During inference all the parameters are assumed to be unconstrained. Results are reported in figure 8b,c, suggesting that our proposed method is able to recover the true parameters of the complex eight-state model. Indeed the scatter plot of figure 8b shows that the estimated parameters are close to the true values identified by the grey diagonal line. Furthermore, results are confirmed in terms of model fit of figure 8c, showing a high level of predictions of the first two moments of the synthetic current trace.

### (g) Comparison with Kalman filter approaches

We compared our proposed inference method with standard methods for stochastic models based on Kalman filter approaches [30,32]. Given that the currently available Kalman filter frameworks of ion channel gating are tailored to describe ligand-gated (but not voltage-gated) dynamics, we developed and implemented an extended Kalman filter (EKF) formulation of stochastic models of voltage-gated ion channel dynamics. Details of our Kalman filter framework are reported in electronic supplementary material, section K. We compared the two methods with a simulation study. In particular, we used the simulation scheme of §2*d* to generate N=100 synthetic current traces of η channels from the true generative data process of the five-state model under the simulation setting of §3*a*. Synthetic data were generated under a total number of ion channels equal to η=1000 and a measurement error variance $\sigma^{2}=10^{-5}{\text{ nA}}^{2}$. The values for the dynamic parameters θθ, the total membrane conductance g, and the rates γ and δ of the flickering mechanism used for the simulations are listed in §3*a*.

**Figure 9.**
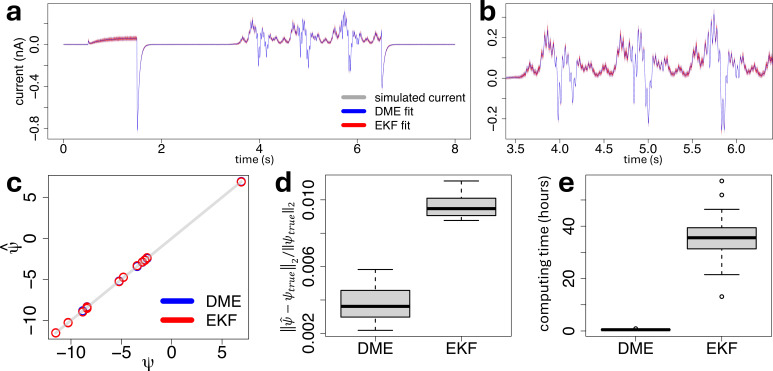
(a): A synthetic current trace (grey line) simulated from the true generative data process of the five-state model under the simulation setting of §3*a* using the dynamic parameters of table 1, under a total number of ion channels η=1000 and a measurement error variance $\sigma^{2}=10^{-5}{\text{ nA}}^{2}$, together with the corresponding model fit, in terms of the first two moments defined by equation (2.23), obtained with either our proposed inference method based on the DMEs (blue) from §2*e*, or its Kalman filter counterpart from electronic supplementary material, section K (red). In both cases, the variance is displayed as a 95% confidence interval (shaded area) around the mean (line). (b): A zoom of plot panel (a) on a portion of the sinusoidal part of the voltage-clamp protocol. (c): Scatter plot of the estimated (y-axis) against true (x-axis) parameters, on a log–log scale, obtained with the DME-based (blue) and the EKF-based (red) approaches, independently from the N=100 simulated stochastic traces. (d): Box plots of the relative error between the estimated and true parameters, across the N=100 simulated stochastic traces, obtained with the DME-based and the EKF-based approaches, where $\|\cdot {\|}_{2}$ is the Euclidean norm. (e): Box plots of the runtime (hours) that the DME-based and the EKF-based approaches took to obtain the optimal parameters, across the N=100 independent simulations.

Then we used our proposed inference method of §2*e*, and its Kalman filter version of electronic supplementary material, section K, to recover all the parameters, independently from each generated synthetic trace. During inference, all the parameters are assumed to be unconstrained. Results are displayed in figure 9, suggesting that both our inference method of §2*e*, and its Kalman filter counterpart from electronic supplementary material, section K, are able to recover the true first two-order moments of the process and the corresponding parameters, as suggested by figure 9a–c. However, the parameter estimates provided by our method based on the differential moment equations have a lower relative error from the true vector parameter compared with the estimates obtained by the EKF-based method, as indicated by the box plots of figure 9d. Finally, our proposed method based on the DMEs outperforms its Kalman filter counterpart in terms of computing time. Indeed, on average across N=100 synthetic traces, the DME-based method only required ≈0.5 h to reach the optimal parameters, against ≈36 h for the Kalman filter method, as displayed by the box plots of figure 9e. The reason for such extreme difference in computational complexity between the two approaches resides in the fact that our inference method does not require us to update the initial conditions of the differential moment equations (2.18) over time, whereas continuous-discrete Kalman filters integrate the differential moment equations between all consecutive time points, $t_{k-1}$ and $t_{k}$, where the measurements are collected, in order to update the initial conditions of the DMEs, and their sensitivities, using the Kalman gain matrix $K\;K_{k}$ and its partial derivatives $\frac{\partial K\;K_{k}}{\partial \psi_{j}}$ at each update step defined by the electronic supplementary material, equations (K8) and (K9), of the extended Kalman filter algorithm 2 detailed in electronic supplementary material, section K. Note that alternative Kalman filter approaches such as Unscented Kalman Filters (see e.g. [53]) may improve inference but would have the same computational complexity as EKFs [54].

### (h) Neglecting the flickering mechanism

**Figure 10.**
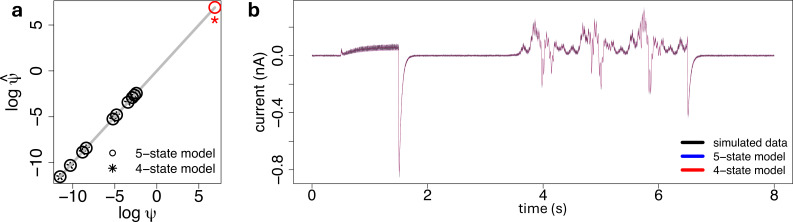
(a): Log–log scatter plot of the estimated against true parameters, obtained after fitting the five-state model (empty dots) and the four-state model (stars) using the proposed inference method. The estimates for the total number of ion channels are highlighted in red. (b): Synthetic ionic current trace (black), and the first two moments of the model fit, defined by equation (2.23), obtained with the four-state (red) and the five-state (blue) formulations, where the variance is displayed as a 95% confidence interval (blue- and red-shaded areas) around the mean (blue and red lines).

We investigated how the parameter estimates are affected when the flickering mechanism is neglected. We simulated synthetic data from the five-state model of figure 2b and calibrated both the true generative five-state model and the four-state model of figure 2a to the simulated traces. We used the Euler–Maruyama method (algorithm 1 of electronic supplementary material, section D1) to simulate stochastic traces xx, obeying the five-state model under the sinusoidal voltage protocol (figure 3a). The values for the dynamic parameters θθ, the total membrane conductance g, and the rates γ and δ of the flickering mechanism used in the state-space model of equations (2.10) and (2.11) for the simulations are listed in §3*a*, and we set the total number of ion channels to η=1000. Then, we used the measurement model of equation (2.11) to simulate the corresponding ionic current trace yy, with $\sigma^{2}=10^{-5}$ ${\text{nA}}^{2}$. Finally, we used our proposed inference procedure from §2*e* to estimate the parameters of both the true generative five-state model and the four-state lumped ($O^{\prime }=O+F$) model, to fit the synthetic ionic current trace yy. During inference all the parameters are assumed to be unconstrained. Results are displayed in figure 10. In particular, figure 10b shows that the fits obtained by calibrating both the four-state and five-state models are very similar, and they almost overlap in terms of mean and variance. Figure 10a shows that if we fit the lumped four-state model to data generated from the true five-state flickering model then $\hat{\eta }$ is an underestimate, but other parameters are inferred well.

To understand this, consider that the four-state model which is over-estimating the true open probability (by calling the lumped $O^{\prime }=O+F$ state ‘open’) will have to under-estimate the true number of channels accordingly to get a similar overall current. If flickering is faster than other gating processes, we expect a quasi-steady equilibrium approximation of the five-state model’s open state to the four-state model’s lumped state to appear, that is


(3.5) $$\frac{O}{O^{\prime }}\approx \frac{O}{O+F}\approx \frac{\gamma }{\gamma +\delta }=\pi_{1}=0.2717...\;,$$

at all times. The computational results confirm that the inferred number of channels scales by approximately the same amount:


(3.6) $$\frac{\hat{\eta }}{\eta_{true}}=\frac{1+\exp{\hat{\phi }}_{9}}{1+\exp\phi_{\text{true},9}}=\frac{261.8557}{1000}=0.2619...\;,$$

where $\psi \;\psi_{true}$ is the vector of the true parameters. So in general, neglecting fast flickering mechanisms will result in underestimating the true total number of ion channels, due to lumped state models overestimating the true open probability, and in turn underestimating the total maximal conductance $g=g_{s}\eta$ by a factor approximately equal to $\pi_{1}.$

Note that the vast majority of deterministic ion channel models ignore flickering by lumping (O+F) states. This is perfectly safe for deterministic fits and predictions where the number of discrete channels and single-channel conductance do not feature, because the product of maximal conductances and open probabilities used in overall conductance calculations will be identical, as in the example above, with maximal conductances scaled by $\pi_{1}$ and open probabilities scaled by $1/\pi_{1}$. So if using macroscopic data to estimate the number of channels (using $\eta =g/g_{s}$, the estimated maximal conductance divided by an independently measured single-channel conductance, as done in e.g. [9,55]), one will have an underestimate in the channel number unless explicitly considering or correcting for the presence of any known flickering states. For hERG, we expect this approach could lead to estimating only $\pi_{1}\approx 27\%$ of the true number of channels. Modellers should therefore always explicitly consider flickering when: (i) examining noise due to stochastic gating; (ii) interpreting the maximal conductance in a model or experiment as the product of the number of channels and single channel conductance; or (iii) interpreting lumped macroscopic open probability as the literal probability of any single channel being open. Points (ii) and (iii) will only be reasonable assumptions when any known flickering behaviour is either included or corrected for.

### (i) Analysing experimental whole-cell voltage-clamp data

**Table 3.** Estimates for each parameter (rows) on a log-scale, obtained after fitting the five-state model to the ionic current traces recorded from each cell (columns). Last row shows the total conductance $g=\eta g_{s}$ .

Cell #	1	2	3	4	5	6	7	8	9
$\log\;\theta_{1}$	−8.52	−8.07	−7.55	−7.35	−8.39	−7.38	−7.50	−8.01	−7.48
$\log\;\theta_{2}$	−2.83	−2.71	−2.76	−2.85	−2.66	−2.73	−3.04	−3.10	−3.14
$\log\;\theta_{3}$	−9.54	−9.45	−9.86	−9.74	−10.26	−8.96	−9.62	−9.87	−8.98
$\log\;\theta_{4}$	−3.01	−3.00	−2.95	−2.96	−2.91	−3.27	−3.09	−3.02	−3.12
$\log\;\theta_{5}$	−2.27	−2.52	−2.00	−2.42	−2.45	−2.52	−2.77	−2.63	−2.72
$\log\;\theta_{6}$	−4.28	−5.90	−4.67	−5.15	−4.71	−4.10	−4.74	−5.05	−5.86
$\log\;\theta_{7}$	−5.58	−5.32	−5.56	−5.15	−5.28	−4.70	−5.23	−5.12	−5.62
$\log\;\theta_{8}$	−3.32	−3.43	−3.28	−3.43	−3.46	−3.68	−3.45	−3.42	−3.37
$\log(g_{s}\times 10^{-6})$	−13.90	−13.91	−13.91	−13.91	−13.91	−13.91	−13.91	−13.90	−13.91
$\log\;\sigma^{2}$	−7.36	−7.40	−6.79	−6.79	−6.91	−8.45	−5.12	−6.40	−7.50
log⁡(η−1)	13.21	12.81	12.92	12.61	13.33	11.39	13.35	12.91	12.24
g	0.50	0.33	0.37	0.27	0.56	0.08	0.57	0.37	0.19

**Figure 11.**
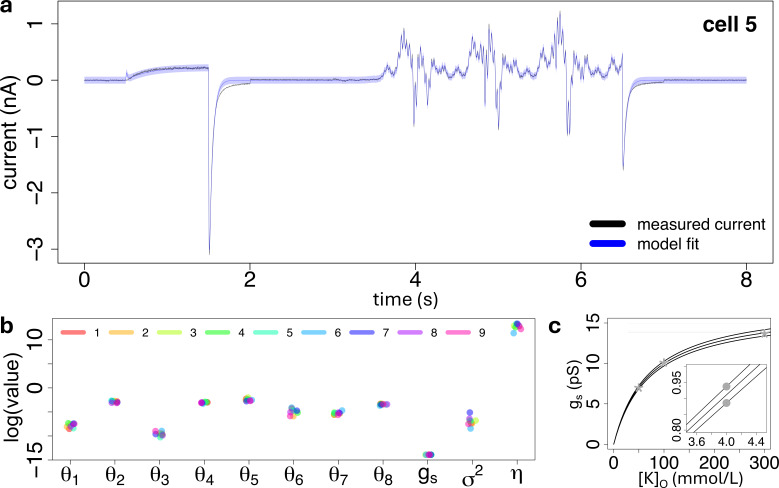
(a): Ionic current trace recorded from cell 5 (black line) and the first two moments, defined by equation (2.23), obtained by fitting the five-state model, where the variance is displayed as a 95% confidence interval (blue-shaded area) around the mean (blue line). Data of the remaining cells and the corresponding model fits are displayed in figure 12. (b): Estimated logarithmic parameters for each cell (colours), whose values are reported in table 3. (c): Data (grey stars) of single-channel conductance (y-axis) observed at three levels of extracellular potassium concentration (x-axis), consistent with [49], and the corresponding fit of the Michaelis–Menten model defined by equation (2.28), including a zoom around the observed extracellular potassium concentration (bottom-right corner). The grey full dots indicate the estimated lower and upper bounds of the single-channel conductance, obtained with equation (2.29) from §2*g*.

**Figure 12.**
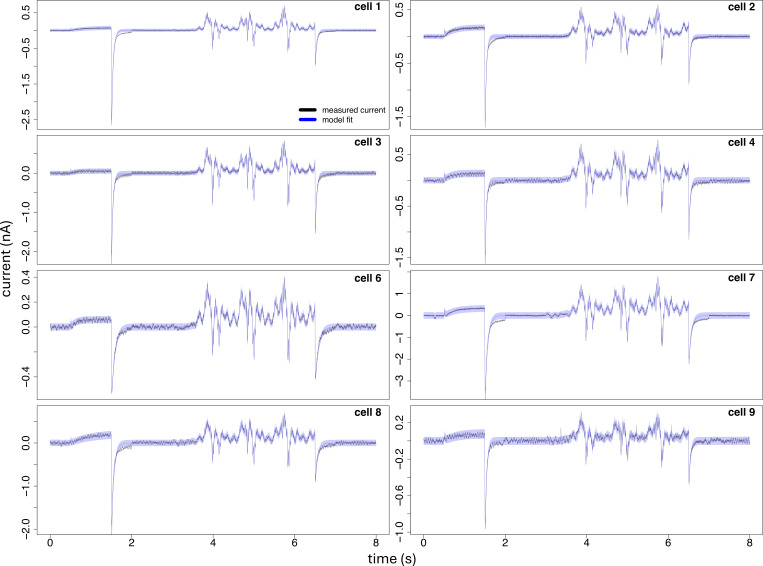
Each plot panel shows the ionic current trace recorded from each cell, and the first two moments, defined by equation (2.23), obtained by fitting the five-state model, where the variance is displayed as a 95% confidence interval (blue-shaded area) around the mean (blue line). Parameter estimates for each cell are displayed in figure 11b and reported in table 3. Data of cell 5 and the corresponding model fit are displayed in figure 11a.

We analysed whole-cell voltage-clamp data recorded from nine CHO cells, stably expressing hERG1a (Kv11.1) at room temperature, under the sinusoidal voltage protocol described in §2*a*. The recorded ionic current traces are displayed in figures 11a and 12. Full details including cell culture, solutions and equipment settings can be found in the original study [12]. We applied our inference method of §2*e* to estimate the vector parameter of equation (2.20) of the five-state model to fit each cell current trace separately. The reaction rates of the flickering mechanism were assumed to be known from single-channel experiments and were set to γ=1/6.7 ms and δ=1/2.5 ms, consistent with §2*f*. Single-channel conductance $g_{s}$ was subject to the box-constraints of equation (2.29), where the single-channel conductance saturation level $g_{s,max}$ and the extracellular potassium concentration at half maximal conductance $[K{]}_{\text{50\%}}$ were estimated from the Kiehn *et al.* experimental data [49], by applying the inference procedure described in §2*g*. We used these box-constraints to make sure that the single-channel conductance $g_{s}$ took only physiologically plausible values, suggested by the experimentally observed extracellular potassium concentration $[K{]}_{o}=4\;\text{mM}$.

Results of the model fitted to the current traces for each cell are displayed in figures 11a and 12, and the corresponding inferred parameters are reported in figure 11b and table 3. The fitted Michaelis–Menten curve of equation (2.28), and the data provided by [49] are displayed in figure 11c. The fitted single-channel conductance saturation level and the extracellular potassium concentration at half maximal conductance are given by


(3.7) $${\hat{g}}_{s,max}=17.02\;\text{pS}\;,\;[\hat{K}{]}_{1/2}=70.72\;\text{mM}\;,$$

respectively. The corresponding lower and upper bounds for the single-channel conductance $g_{s}$ were computed accordingly, following equation (2.29) of §2*g*, as


(3.8) $$g_{s,lb}=0.886\;\text{pS}\;,\;g_{s,ub}=0.938\;\text{pS}\;.$$

Figure 11b and table 3 suggest that the values of the estimated dynamic parameters θθ and the single-channel conductance $g_{s}$ are similar across different cells, and the corresponding model predictions accurately describe the first two moments of the ionic currents, as shown in figures 11a and 12. Our proposed state-space model formulation of ion-channel gating of equations (2.10) and (2.11) and the inference procedure of §2*e* allowed us to distinguish between noise due to either measurement error or stochastic gating, reflected by the variability in the estimated variance of the measurement noise ($\sigma^{2}$) and the total number of channels (η) across the cells, as suggested by the values reported in figure 11b and table 3. Finally, our measurement model formulation of equation (2.11) allowed us to identify the source of variability in the total cell conductance $g=\eta g_{s}$ across the cells, as a result of the high variability in the total number of channels η in the cell membrane, and the low variability in the estimated single-channel conductance $g_{s}$.

## 4. Discussion

We have proposed a method to calibrate stochastic models of ion channel gating to whole-cell voltage-clamp macroscopic data. To this end, we first introduced a state-space model, whose dynamic component is a stochastic reaction network describing the time-changes in the proportion of channels xx being in a particular configuration. The measured ionic current yy is linked to the underlying ion channel configurations xx via an Ohmic expression, having an iid measurement error. Then, we extended a four-state ion channel model of hERG gating [12] by introducing an additional Markov state describing a non-conducting open configuration, motivated by experimental findings [48]. Subsequently, we proposed a method to simulate synthetic data from a given stochastic reaction network of ion channel gating. Finally, we introduced an inference method to estimate the parameters of a stochastic ion channel model written in our proposed state-space formulation. The inference scheme is based on a maximum likelihood approach, aimed at minimizing the negative log-likelihood, having a Gaussian distribution whose mean and variance are obtained by solving the differential moment equations of the stochastic process xx. This means that the likelihood that has to be optimized only depends on the first two moments of the process xx, but not on the process itself, for which computationally expensive filtering techniques would be required instead [33,37,38].

Simulation studies suggest that our proposed inference method is able to recover the true parameters of a stochastic reaction model of ion channel gating with low uncertainty, and better distinguishes between measurement error and stochastic noise, compared with inference approaches that are based on deterministic models of ion channel gating. Furthermore, results from simulations indicate that our proposed inference method: (i) is robust against uncertainty induced by sampling frequency issues that may be related to unobserved measurements due to experimental limits; (ii) scales well to complex structures of ion channel gating; (iii) outperforms inference methods based on Kalman filters. Our inference method does not only provide more accurate parameter estimates, but it is also computationally efficient, being approximately 72× faster, in our simulation setting, than the extended Kalman filter approach. This difference in computational complexity between the two approaches can be easily attributed to the fact that our proposed inference method does not require the updating formulas of electronic supplementary material, equations (K8) and (K9), for correcting the initial conditions of the DMEs, that characterize Kalman filter approaches. In particular, for a p-dimensional vector parameter, such equations feature (4+p×8) matrix sums, (8+p×17) matrix multiplications and one matrix inversion, that must be computed between every pair of consecutive time points $t_{k-1},t_{k}$, (k=1,…,τ), where the measurements were collected. Therefore, approaches that are based on Kalman filters become impractical for the analysis of ionic current traces obtained from voltage-clamp experiments, typically having a sampling frequency on the order of 10 kHz.

Also, an additional simulation study where we used our proposed five-state model of ion channel gating as a true generative process, and the four-state model as a candidate model, suggested that across all the unknown parameters the total number of channels η is the most affected by the misspecification of the flickering mechanism, even if both models provided similar goodness of fit. This means that modellers should consider the flickering mechanism when calibrating models of ion channel gating to fit whole-cell voltage-clamp data, in order to obtain unbiased estimates for η and, in turn, for the total conductance $g=g_{s}\eta$ of the cell membrane. Finally, the application of our inference method to whole-cell voltage-clamp data collected from nine CHO cells showed that the five-state model is able to describe the dynamics of ion channel gating from the experimentally observed ionic current measurements, and that the parameter estimates are comparable across cells. Also, our proposed inference method allowed us to unveil the heterogeneity in the maximal conductance g across cells, as a function of the total number of channels η in the cell membrane, as suggested by the parameter estimates.

Note that there is a small amount of 50 Hz ‘mains hum’ visible in figure 12 on some of the experimental data (particularly cells #4, 6, 8, 9). We were initially concerned that this would be interpreted as stochastic gating, but the wider confidence regions for these cells where current is approximately zero (at the beginning and end of the protocol) show that it is instead being incorporated into the measurement error (ε in equation 2.11). This behaviour is thought to be due to 50 Hz measurement noise being consistent throughout the trace, as ε is, and so its presence is not adversely impacting inferences based on additional variance due to stochastic gating.

Results from this work should improve data-driven models of ion channel gating by accounting for stochastic noise and measurement error during inference [56]. Our proposed framework could be used to explore the experimentally observed random patterns of ion channel gating [3] and their effects on the electrophysiological dynamics of the whole cell [5,6,10], such as, in cardiac myocytes, the behaviours of beat-to-beat variability, the formation of early after depolarizations (EADs), or potassium channel block [9,55,57]. However, other sources of variability might affect the parameter estimates besides stochastic noise and measurement error, such as experimental artefacts [25]. An extension of our state-space formulation and the companion inference procedure accounting for this additional source of error is the goal of our future research. Although our framework is tailored to analyse whole-cell voltage-clamp data, a slight modification can allow us to analyse different types of patch-clamp data, such as single-channel measurements. Applications in alternative contexts of population dynamics besides ion channel gating, where partially observed time-inhomogeneous stochastic processes are affected by multiple sources of error, could also be explored with this approach.

## Data Availability

Data and code to replicate this study is openly available at [58]. A permanently archived version of the code is openly available at [59]. Supplementary material is available online [60].
